# Solving the Problem of Assessing Synergy and Antagonism for Non-Traditional Dosing Curve Compounds Using the DE/ZI Method: Application to Nrf2 Activators

**DOI:** 10.3389/fphar.2021.686201

**Published:** 2021-06-07

**Authors:** Elizabeth M. Repash, Kaitlin M. Pensabene, Peter M. Palenchar, Aimee L. Eggler

**Affiliations:** Department of Chemistry, Villanova University, Villanova, PA, United States

**Keywords:** synergy, antagonism, Nrf2 pathway, Keap1 pathway, non-traditional dosing curves, hormesis, Loewe Additivity

## Abstract

Multi-drug combination therapy carries significant promise for pharmacological intervention, primarily better efficacy with less toxicity and fewer side effects. However, the field lacks methodology to assess synergistic or antagonistic interactions for drugs with non-traditional dose response curves. Specifically, our goal was to assess small-molecule modulators of antioxidant response element (ARE)-driven gene expression, which is largely regulated by the Nrf2 transcription factor. Known as Nrf2 activators, this class of compounds upregulates a battery of cytoprotective genes and shows significant promise for prevention of numerous chronic diseases. For example, sulforaphane sourced from broccoli sprouts is the subject of over 70 clinical trials. Nrf2 activators generally have non-traditional dose response curves that are hormetic, or U-shaped. We introduce a method based on the principles of Loewe Additivity to assess synergism and antagonism for two compounds in combination. This method, termed Dose-Equivalence/Zero Interaction (DE/ZI), can be used with traditional Hill-slope response curves, and it also can assess interactions for compounds with non-traditional curves, using a nearest-neighbor approach. Using a Monte-Carlo method, DE/ZI generates a measure of synergy or antagonism for each dosing pair with an associated error and *p*-value, resulting in a 3D response surface. For the assessed Nrf2 activators, sulforaphane and di-*tert*-butylhydroquinone, this approach revealed synergistic interactions at higher dosing concentrations consistently across data sets and potential antagonistic interactions at lower concentrations. DE/ZI eliminates the need to determine the best fit equation for a given data set and values experimentally-derived results over formulated fits.

## Introduction

The field of drug discovery is looking beyond the traditional one-target, one-drug paradigm (Keith et al., 2005). Combining two or more drugs in a single treatment has significant promise for pharmacological intervention, due primarily to better efficacy, less toxicity, and fewer side effects (Lehàr et al., 2009). This approach has been successfully adopted in diverse areas including chemotherapy (Mokhtari et al., 2017), malaria (Fidock et al., 2004), and HIV (Arts and Hazuda, 2012)*.* The central question is whether two drugs interact synergistically—that is, if their combined effect is greater than what is predicted based on their individual effects—and this seemingly simple question has been challenging to address. For over 35 years the field has actively formulated and evaluated various methodologies to assess synergism and its counterpart, antagonism, and numerous reviews discuss the appropriate use of these methods [e.g., (Geary, 2013; Foucquier and Guedj, 2015; Caesar and Cech, 2019)]. Currently, the field lacks a method to assess interactions for compounds with dosing curves that are not easily fit to a Hill-slope equation.

**FIGURE 1:**
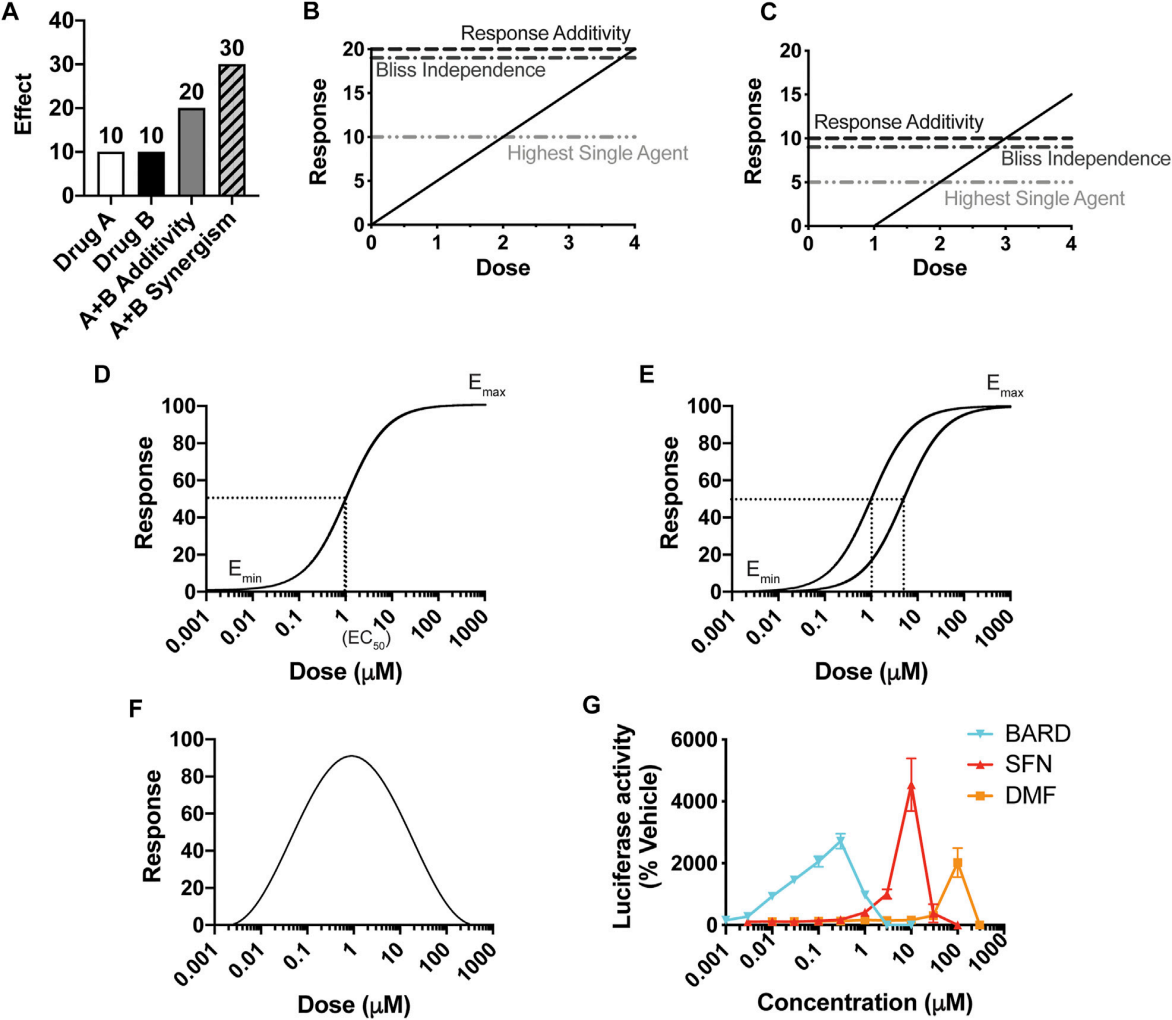
The importance of a dose response curve, and its overall shape, for the prediction of synergy. **(A)** The definition of synergy by the effect-based Response Additivity method, where the predicted additive effect is the sum of the effects of Drug A and Drug B, and actual effects that are greater than this sum are synergistic. **(B, C)** The Sham Combination test for three common “effect-based” methods, for which the effects of single doses determine the predicted additive effect. A single drug plays the role of both Drug A and Drug B, and thus the actual additive effect is known; here it is the effect of a dose of (2 + 2) = 4, with a corresponding response of 20 in **(B)** and 15 in **(C)**. Only for **(B)**, in which *y* = mx, does the predicted additive effect match the actual one. **(D)** A classic Hill-slope curvilinear fit, which approaches a maximum effect. **(E)** Two Hill-slope curves with a “constant potency ratio,” which requires equivalent Hill-slopes (parallel curves on a log-scaled *x*-axis) and the same effect at 100% response. **(F)** A hormetic dosing curve, which has a biphasic effect, here shown as decreasing at increasing concentrations. **(G)** Dose-response curves for three Nrf2/ARE activators in clinical use or in clinical trials.

To assess whether any two drugs interact, either with synergism or antagonism, the central objective is to determine the predicted additive effect of the combination treatment (i.e., the effect if there is zero interaction between the drugs). Then, if that predicted additive effect is different than the actual effect of the combination treatment, by definition the drugs interact. While determining a predicted additive effect at first appears to be a straightforward task, decades of intense discussion in the field show otherwise. A common approach to determine the predicted additive effect of a combination treatment of drug A and drug B for a particular dosing pair (a,b) is to add the individual effects (Figure 1A). However, this method, Response Additivity, only works under highly specific conditions, as illustrated by the Sham Combination test (Figures 1B,C): when the dosing curves are linear with zero intercepts. In the hypothetical situation of the test, the two drugs A and B have identical dosing curves. Thus, the effect of the dosing pair (a,b) = (2,2) is known—it will be the effect for a dose of 4. As shown in the example in Figure 1B, a line with zero intercepts passes the Sham Combination test; the predicted additive effect for (2,2) is 10 + 10 = 20, which matches the actual effect for the dose of 4. However, any other line or curve fails the test, including, for example, a straight line with an *x*-axis intercept greater than zero (e.g., Figure 1C, where a dose of 4 would give an effect of 15). Drugs rarely have linear dosing curves with zero intercepts; commonly they fit to a Hill-slope type curve (Figure 1D) (Caudle and Williams, 1993).

The other two widely-used methods that consider the effects of the individual treatments at single doses are Highest Single Agent (HSA) and Bliss Independence. HSA is the simplest approach and asserts that if the combined effect is larger than the maximal effect of either drug alone, then the drugs act synergistically. In the Bliss Independence method, a probabilistic model for drug interactions is assumed (E_A_ + E_B_ – E_A_E_B_), and the effects are expressed as a probability (greater than 0 but less than 1). For example, expressing the effects in Figures 1A,B as probabilities out of 100 generates 0.1 for E_A_ and E_B_, a predicted additive probability of 0.1 + 0.1 – 0.01 = 0.19, and thus a predicted additive effect of 19. These methods, reviewed extensively elsewhere, also fail the Sham Combination test (Figures 1B,C), present other limitations, and are rarely valid for a given combination of drugs (Goldoni and Johansson, 2007; Geary, 2013; Foucquier and Guedj, 2015).

The alternative to effect-based approaches is to calculate a predicted additive effect by using the predictive value of the individual dosing curves [reviewed in (Foucquier and Guedj, 2015)]. The fundamental principles for this “dose/effect” approach are known as Loewe Additivity, first introduced in the 1870s (Fraser 1871, 1872), and then formally defined by Loewe in the 1920s and developed further by him in the 1950s (Loewe and Muischnek 1926; Loewe, 1927, Loewe 1953, Loewe 1959). Its principles are detailed in the section titled, *Loewe Additivity Principles Behind the DE/ZI Method and the Benefits of Releasing the Constraint of a Constant Potency Ratio*. Loewe Additivity is the most-widely accepted approach in the field, surviving almost a century of dissection (Greco et al., 1995). The effort in the field to utilize the Loewe Additivity principles has largely focused on drugs with traditional dose response curves, which approach a maximum effect and fit to a Hill-slope equation (Figure 1D). Loewe Additivity is the basis for the widely used approaches of the Combination Index (Foucquier and Guedj, 2015), isobologram analysis (Greco et al., 1995), and the median-effect approach of the Chou-Talalay method (Chou and Talalay, 1984). The Chou-Talalay method has been used to evaluate synergy and antagonism in ∼5,000 published studies in large part due to the software products CompuSyn (now freely available) and Calcusyn, a commercial product. However, as summarized below and shown in depth by others, the Chou-Talalay method is only appropriate if the dosing curves being analyzed fit to a Hill-slope equation and have a constant potency ratio Figure 1E, which is often not the case for the drugs they are being using to analyze (Tallarida, 2007; Calabrese, 2008b; Geary, 2013).

**FIGURE 2:**
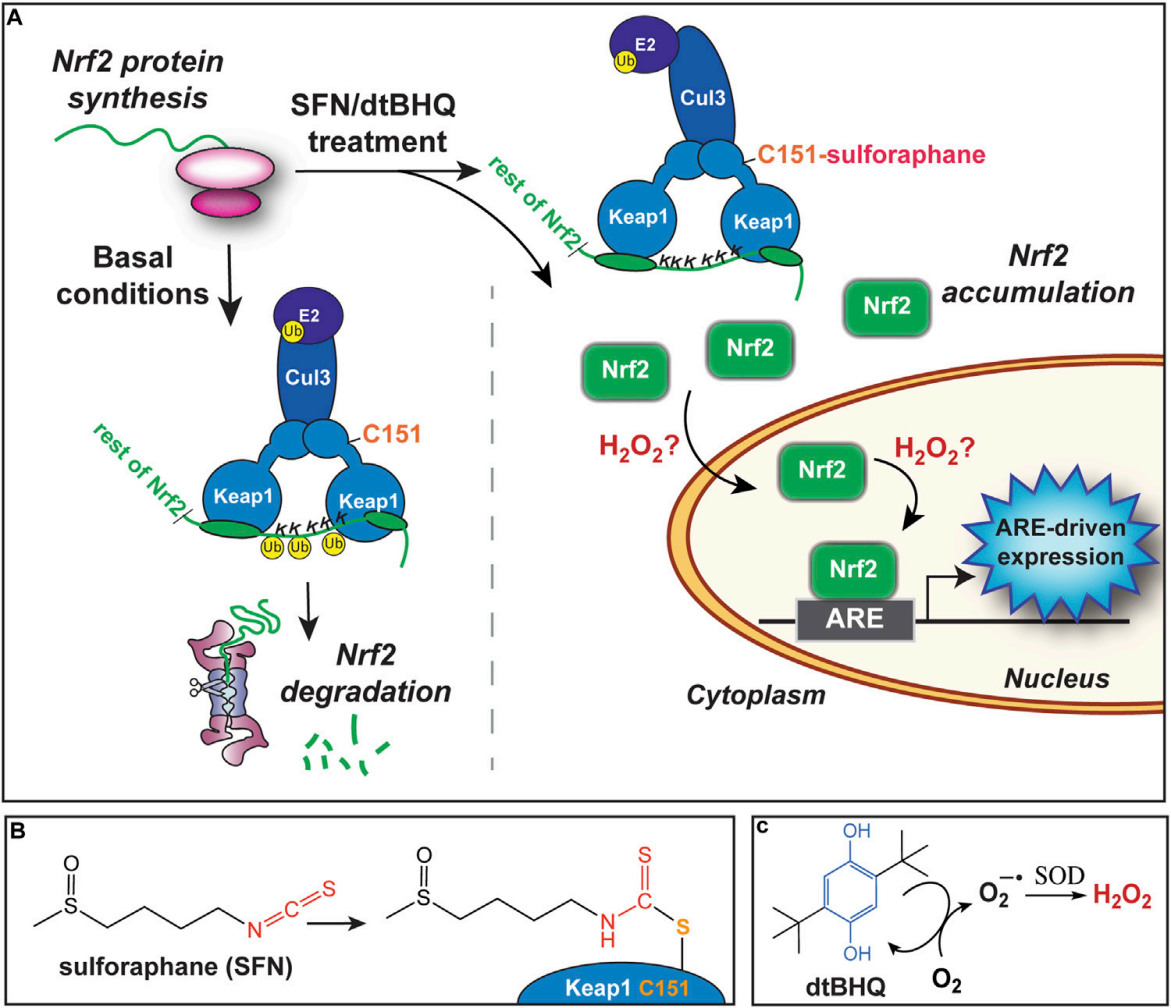
Activation of the Nrf2/ARE pathway by sulforaphane and dtBHQ. **(A)** Nrf2 protein is constitutively synthesized. Under basal conditions, Nrf2 is targeted for ubiquitination and degradation by the Keap1 protein, which forms a bridge between Cul3 and an E2 ligase and Nrf2, positioning Nrf2 lysines for ubiquitination. Upon treatment, sulforaphane modifies Keap1 C151, also shown in **(B)**, altering the Keap1-Cul3 conformation and preventing ubiquitination of Nrf2 lysines. Newly synthesized Nrf2 accumulates. Subsequently, Nrf2 translocates to the nucleus and binds to copies of the ARE upstream of cytoprotective genes. The inclusion of dtBHQ generates H_2_O_2_, also shown in **(C)**, which acts downstream of Nrf2 protein accumulation, possibly by increasing nuclear translocation or enhancing Nrf2’s induction of ARE-driven gene expression. Possible mechanisms are further described in the Discussion. **(B)** The structure of sulforaphane at left, and in conjugation with Keap1 C151 at right. **(C)** The structure of dtBHQ, which redox cycles to donate electrons to dioxygen and generate superoxide. Superoxide dismutase (SOD) converts this to hydrogen peroxide.

Our primary goal is to evaluate the interactions of small molecule activators of the transcription factor Nrf2. Nrf2 regulates hundreds of genes, many with cytoprotective functions including detoxification, repair, and the oxidative stress response, through binding to its cognate antioxidant response element (ARE). The Nrf2/ARE pathway is activated by both electrophiles and oxidative stress. A major target of reactive species are cysteines on the Keap1 protein, a primary Nrf2 repressor that targets Nrf2 for ubiquitination and degradation (Figure 2A). Keap1 has 25 conserved cysteines, 11 of which comprise the sensor system (Suzuki et al., 2019). Numerous phytochemical small molecule activators have been identified (Eggler and Savinov, 2013), with a number of them progressing to clinical trials. For example, sulforaphane, found in high concentrations in broccoli sprout extracts, is the subject of over 70 clinical trials. Sulforaphane targets Keap1 C151 (Figures 2A,B), halting Nrf2 ubiquitination and allowing Nrf2 to accumulate (Zhang and Hannink, 2003). Given the clinical promise of Nrf2/ARE pathway activation by phytochemicals and engineered small molecules (Al-Sawaf et al., 2015; Houghton et al., 2016; Staurengo-Ferrari et al., 2019; Tu et al., 2019), there is significant interest in finding synergistic combinations of Nrf2 activators, and avoiding antagonistic interactions in formulations.

Thus far, Nrf2/ARE activators almost universally have a hormetic dose response, with a characteristic U- or V-shaped curve (Figure 1F). For example, sulforaphane, bardoxolone methyl, and dimethyl fumarate each show hormesis for activation of the Nrf2/ARE pathway (Figure 1G, adapted from (Copple et al., 2014)). Dimethyl fumarate, marketed as Tecfidera^®^, is FDA-approved for multiple sclerosis (Mills et al., 2018). Bardoxolone methyl is currently in clinical trials as a potential therapeutic for several forms of chronic kidney diseases and varied other conditions, including COVID-19 (Reata Pharmaceuticals, 2021; U.S. National Library of Medicine, 2021). Other examples of hormetic Nrf2 activators include the endogenous electrophile 15dPGJ2 and iodoacetamide (Ricart et al., 2009). A variety of other drugs exhibit hormetic dose-response curves, including those with anxiolytic (Calabrese, 2008a), anti-tumor (Paoletti et al., 1990), and anti-seizure (Calabrese, 2008c) actions. Hormesis is well documented in the toxicology field in particular (Calabrese and Blain, 2005). Phytochemicals that are produced by plants for their defense (e.g., sulforaphane) are generally toxic to humans at high doses but have beneficial effects at lower doses, including Nrf2/ARE activation (Calabrese et al., 2010). Few studies have considered how to predict an additive effect for compounds with hormetic dosing curves (Calabrese, 2008b).

Synergistic interactions of Nrf2 activators have been reported by several groups. Response Additivity has been used to assess interactions of sulforaphane and selenium (Li et al., 2012) as well as components of the commercially available supplement Protandim (Velmurugan et al., 2009). Approximately ten different phytochemicals have been evaluated in various combinations using the Chou-Talalay method (Saw et al., 2010; Saw et al., 2011; Saw et al., 2012; Saw et al., 2014). These methods of assessing interactions have not been evaluated for their usefulness for Nrf2/ARE activators, in particular with respect to hormetic dosing curves.

Previously, we observed what qualitatively appeared to be a strong synergistic interaction in HaCaT keratinocyte cells, using a model system in which cells were co-treated with electrophilic sulforaphane and a small-molecule diphenol that generates reactive oxygen species through redox cycling, di-*tert*-butylhydroquinone (dtBHQ) (Bauman et al., 2018) (Figures 2B,C). We hypothesized sulforaphane and dtBHQ would act synergistically, given their distinct chemical natures and thus possibly distinct targets in the Nrf2/ARE pathway. We found that concentrations of dtBHQ that showed little activation of the pathway on their own significantly enhanced sulforaphane’s activation of the pathway, observed both by western blotting and an ARE reporter assay, suggestive of a synergistic activation. There were two other aspects of the data that strongly suggested a synergistic interaction between the compounds, that is, that sulforaphane and dtBHQ-generated ROS have distinct targets in the pathway. First, the maximum ARE reporter activation achieved by the combination treatment was more than twice as high as either compound could achieve on its own. Second, while sulforaphane treatment increased Nrf2 protein levels, as expected based on sulforaphane’s ability to modify Keap1 C151 and promote Nrf2 escape from Keap1 repression (Figure 2A) (Zhang and Hannink, 2003; Hu et al., 2011; Baird et al., 2013; Saito et al., 2016), addition of dtBHQ with sulforaphane did not further increase Nrf2 levels. Thus, as shown in Figure 2, dtBHQ-generated reactive oxygen species do not act on Keap1 cysteines, which would have increased Nrf2 protein levels. Rather, they act downstream on as-yet unidentified targets. In addition to the qualitatively strong synergistic effects observed at most tested concentrations, there were possible indications of antagonism at the lowest tested concentrations of sulforaphane and dtBHQ.

In order to quantitatively analyze the data for interactions between sulforaphane and dtBHQ, we examined available methods for their appropriateness for Nrf2 activators in general and for these compounds in particular. In addition to the non-traditional, hormetic nature of the sulforaphane dosing curve, the dtBHQ curve is also atypical, with a small but reproducible suppression of ARE reporter expression, compared to basal levels, at the lowest tested concentration (Bauman et al., 2018). First, since the compounds do not have linear dose-response curves with a (0,0) *x*,*y* intercept, effect-based methods, including Response Additivity, are not applicable, as shown in Figure 1. An illustrated explanation for the dtBHQ dosing curve is given in Supplementary Figure S1. Looking to dose/effect-based methods, the typical Loewe Additivity approach is not appropriate for many types of data, including ours, for two reasons. First, it requires the dose-effect curve to be fit, or modeled, to a particular equation. In practice, most methods including the Chou-Talalay method fit data to a Hill-slope equation. Other equations can be used to fit the data, but there are limitations to this approach for non-traditional dosing curves, as explained in *The Nearest-Neighbor Approach Alternative to Curve-Fitting*. Second, assuming the data can be reasonably modeled, most methods based on Loewe Additivity require a constant potency ratio, shown in Figure 1E for Hill-slope equations. This constraint is summarized in *Loewe Additivity Principles Behind the DE/ZI Method and the Benefits of Releasing the Constraint of a Constant Potency Ratio* and extensively reviewed elsewhere (Geary, 2013; Foucquier and Guedj, 2015).

Given these limitations, we developed an R code that applies the time-tested principles of Loewe Additivity but releases the constraint that there must be a constant potency ratio for the two dosing curves. Named after the first principles of Loewe Additivity (*Loewe Additivity Principles Behind the DE/ZI Method and the Benefits of Releasing the Constraint of a Constant Potency Ratio*), the Dose-Equivalence/Zero Interaction (DE/ZI) method can be used for data with or without a constant potency ratio. For non-traditional dosing curves, to circumvent the issues associated with fitting a dosing curve to an equation with a reasonable number of parameters (*The Nearest-Neighbor Approach Alternative to Curve-Fitting*), we introduce a nearest-neighbor approach. Other advantages of DE/ZI are that synergy or antagonism are assessed for each dosing pair, allowing complex interactions across the range of concentrations to be captured, and that a *p*-value is generated for each assessed interaction.

## Materials and Methods

### Loewe Additivity Principles Behind the DE/ZI Method and the Benefits of Releasing the Constraint of a Constant Potency Ratio

**FIGURE 3:**
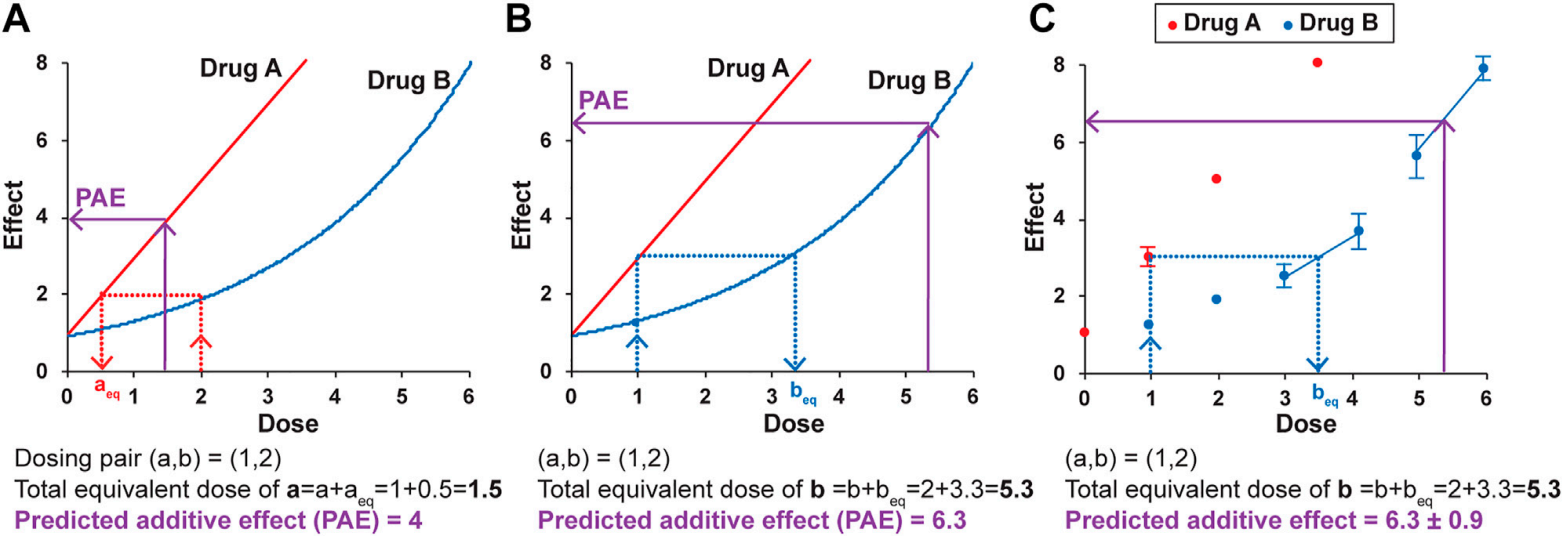
The principles of Loewe Additivity and their application to the DE/ZI method. A predicted additive effect (PAE) is determined by calculating a total equivalent dose using either the dosing curve for drug A **(A)** or drug B **(B)**, and then interpolating using the same dosing curve to determine the predicted effect. See text for details. **(C)** A predicted additive effect is determined using the dose-effect data for Drug B, here with the nearest-neighbor method. The two nearest points to the effect or dose of interest are used for interpolation. The error in the predicted additive effect is generated by a Monte Carlo simulation in the R code, as described in the Methods. The values given here are for illustrative purposes.

Loewe Additivity is based on two primary principles. The first is *dose-equivalence*—that for a given effect of dose b of drug B, there is an equivalent dose of drug A that gives the same effect. The example in Figure 3A illustrates that for a dose of 2 for drug B, with an effect of 2, there is an equivalent dose of drug A (a_eq_) that also has an effect of 2, found by interpolating from the dosing curve for drug A. In the example, a_eq_ is 0.5. The second principle is that *if* there is *zero interaction* between the drugs (if one drug is not impacting the other’s effect), then the dose and the equivalent dose can be added to get a total equivalent dose. The predicted additive effect (PAE) of the combination treatment is thus the effect determined from the total equivalent dose. In the example of dosing pair (a,b) = (1,2) (Figure 3A), the doses of a and a_eq_ added together (1 + 0.5) give a total equivalent dose of drug A of 1.5. The dosing curve for drug A is used to determine the predicted additive effect, which in this example is an effect of 4. If the actual effect of the combination of drug A at a dose of 1 and drug B at a dose of 2 is larger than 4, that is evidence for synergy and an interaction between the two drugs. Any dosing pair with a lower actual effect then the zero interaction-predicted additive effect is antagonistic and also evidence of an interaction between the two drugs. In other words, the null hypothesis in Loewe Additivity is that there is zero interaction, and the actual effect is the same as the predicted additive effect (PAE). If the actual result of the combination treatment is different than the predicted additive effect, the null hypothesis of zero interaction is not supported, and there is evidence of an interaction.

The apparently straightforward nature of this approach becomes less so when one considers interpolating from Drug B’s curve to determine b_eq_ and the resulting predicted additive effect. The major assumption made by the methods based on Loewe Additivity is that *the same predicted additive effect is obtained when interpolating from either dosing curve*. For the example in Figure 3, for the same dosing pair (a,b) = (1,2), a different predicted additive effect of 6.3 is generated when Drug B’s curve is used for interpolation (Figure 3B **)**. This is the inherent nature of the Loewe Additivity approach. The repercussions of this somewhat uncomfortable dual result are detailed elsewhere (Tallarida, 2007; Geary, 2013). In brief, the current widely used versions of Loewe Additivity make the key assumption that the two dosing curves both fit to a Hill-slope type equation with the same Hill coefficient (*n*) and that they come to the same maximum value, making them parallel on a log-dose scale (Figure 1E) (Roell et al., 2017). Under this assumption, there is a “constant potency ratio” *R*, where a single value of *R* is obtained for all dosing pairs:

$$R=\frac{a+a_{\mathrm{eq}}}{b+b_{\mathrm{eq}}}$$

With a constant potency ratio, the PAE determined from the curve for drug A (PAE_A_, from a + a_eq_) is the same as the PAE determined from the curve for drug B (PAE_B_, from b + b_eq_). Mathematically, this is described in the following equation, where for all dosing pairs (a,b):

$$\mathrm{Combination Index}\left(\mathrm{CI}\right)=\frac{a}{a+a_{\mathrm{eq}}}+\frac{b}{b+b_{\mathrm{eq}}}$$

In this model, a CI value of 1 results when drug A and drug B have no interaction and the effects are simply additive. A synergistic interaction is indicated by CI < 1, and for antagonism, CI > 1. As noted by others, this equation is equivalent to the Chou Talalay combination index equation (e.g., (Geary, 2013; Roell et al., 2017)).

This particular dosing curve scenario (PAE_A_ = PAE_B_) for drug-drug interactions is often graphically represented by isobolograms, in which a plot of the dose of drug A required for a certain effect vs. the dose of drug B required for that effect fits to a straight line (Greco et al., 1995). Any point along this line is indicative of additivity, any point below indicates synergy, and any point above indicates antagonism. The widely-used methods based on Loewe Additivity—the Combination Index and the Chou-Talalay method—require for two given drugs a constant or at least reasonably constant potency ratio, and thus a linear isobologram.

However, the potency ratio is often not constant for two drugs under consideration (Grabovsky and Tallarida, 2004; Geary, 2013; Lederer et al., 2018). A non-constant potency ratio results in two predicted additive effects for each dosing combination, as illustrated in Figures 3A,B. Accordingly, there will be two curved isoboles—two apparently equally valid, but different, predicted results. Loewe himself noted this was a likely outcome (Loewe, 1953). As posited by others, the fact that the linear isobole assumption fails for many drugs may have gone largely ignored due to the metaphorical descriptions Loewe used (Tallarida, 2007), or perhaps due to influential reviews in the field in support of the linear isobologram whose mathematical bases have since been shown to contain errors (Geary, 2013). Others have developed models for interaction analysis that allow for multiple isoboles (Grabovsky and Tallarida, 2004). However, these models are also specific for data that fit to a Hill-slope equation, and as such they are not appropriate for data that do not fit well to this equation. In sum, most existing applications of Loewe Additivity’s principles, including the widely-used Chou-Talalay method, incorporate a constraint—that there must be only one predicted additive effect. The DE/ZI method releases this constraint and calculates both predicted additive effects that result from Loewe Additivity.

### The Nearest-Neighbor Approach Alternative to Curve-Fitting

An issue for analyzing interactions of hormetic compounds in particular is the difficulty in fitting them to an equation. To fit them properly, these compounds require dosing curves with many points, and equations with a high number of powers of freedom (Zou et al., 2013). Obtaining sufficient data points to fully define a hormetic dosing curve can be cumbersome, in particular when working with compound libraries or rare samples. Moreover, the biphasic nature of hormesis can greatly complicate analysis of synergistic interactions. By definition, a given effect is observed at two distinct doses of a hormetic drug (Figure 1F). However, the problem can be simplified if the region of interest in the dosing curve is the initial portion with the lower doses. For Nrf2/ARE activators and hormetic compounds in general, higher concentrations that lead to a decrease in effect also cause toxicity (Calabrese et al., 2010; Copple et al., 2014) and while important to note in drug development, are not in the range relevant for pharmaceutical dosing. Therefore, in the DE/ZI method, only the data for the lower-dose half of a hormetic curve (for Nrf2/ARE activation, the upward-trending effects) are used.

In regard to fitting this left-most half of the dosing curve for hormetic data, while a linear or Hill-slope type equation might reasonably fit the given data for any particular Nrf2 activator, it can be time-consuming to test and evaluate various fits to determine whether a given fit is indeed reasonable. In addition, the chosen fit may be arbitrary, i.e., without a biological basis. In general, we wanted a method that was more broadly applicable to any given set of dosing data. Here, we introduce a “nearest neighbor” approach. An example is shown in Figure 3C. The scenario is analogous to that in Figure 3B, but instead of a fitted curve, each individual data point is used in the analysis. For example, to determine b_eq_ for a dose of A of 1 with an effect of 3, the two points on the b dosing “curve” whose effects flank the effect of 3 on the *y*-axis are selected and used to generate an equation of the line between them. This equation is then used to solve for b_eq_. Similarly, the predicted additive effect is solved from the equation of the line fit to the points that flank the total equivalent dose of B on the *x*-axis. (If needed, the effect of a dose of 1 for drug A can also be determined using the same method even if that dose itself was not tested, choosing points on the A dosing curve that flank 1 to interpolate for the expected effect.)

One caveat of using the nearest-neighbor approach for hormetic compounds in DE/ZI is that it can underestimate the extent of synergy for high actual effects of combinations, specifically, those that lie above the highest point on the individual dosing curve. This is because these necessarily cannot be interpolated between two points on the actual dosing curve. To accommodate this, DE/ZI extrapolates the predicted additive effect using the line formed by the point on the dosing curve with the highest actual effect and the point for the next lowest dose. In reality, a hormetic dosing curve decreases in effect after reaching its peak. Therefore, the predicted additive effect calculated by DE/ZI may be quite a bit higher than one determined from the whole hormetic dosing curve, leading to an underestimation of the magnitude of the FoldSynergy value for these combinations. However, dosing combinations that result in an effect higher than any on the individual dosing curve will still be found to be synergistic.

### Generating a Data Set to Analyze Interactions of Sulforaphane and dtBHQ for Nrf2/ARE Activation

#### Cell Culture

The HaCaT cell line (Cell Line Service; L#300493-4212) was maintained at 37°C in 5% CO_2_ in phenol red-free and sodium pyruvate-free high-glucose Dulbecco’s modified Eagle’s medium (DMEM) supplemented with 10% fetal bovine serum, 15 mM HEPES (4-(2-hydroxyethyl)-1-piper-azineethanesulfonic acid) pH 7.2, and 4 mM L-glutamine (referred to as complete media). Complete media was stored in the dark at 4°C, with aliquots heated to 37°C just prior to use. Cells were maintained between 50 and 80% confluency during both propagation and experiments, passaging every three days. When designing plate layouts for experiments, care was taken to avoid any neighboring well effect of the treatments with dtBHQ, as previously reported for tBHQ (Braeuning et al., 2012). Thus, a separate 24-well plate was used for the DMSO (vehicle) and sulforaphane-only wells, and titrations of dtBHQ were set up from lowest to highest concentration across a plate.

#### Dual Luciferase Antioxidant Response Element Reporter Assay

Cells were plated in a 24-well plate at 5.0 × 10^4^ cells/well in 500 µl of complete media and allowed 18 h to recover. Following recovery, cells were co-transfected with two luciferase reporters, 15 µg/well of the Renilla luciferase reporter plasmid, pRL-TK (Promega), and 45 µg/well of firefly luciferase ARE reporter pGL4.37 (Promega). Per manufacturer’s instructions, plasmids were incubated for 20 min with 50 µl/well of Opti-MEM^™^ reduced serum medium with 1 µl/well of TransIT^®^-2020 (Mirus Bio). Cells were incubated at 37°C for 4 h with transfection mix prior to replacing the media with 1 ml of fresh complete media. The following day, immediately prior to treatment, spent media was replaced with 2 ml of fresh complete media. Treatments were added directly to each corresponding well. Vehicle for sulforaphane and dtBHQ was DMSO, and total DMSO was equal across all treatments and did not exceed 0.05% DMSO (v/v). Cells were harvested 18 h post-treatment by rinsing with phosphate buffered saline and then lysed with 100 µl/well of Passive Lysis Buffer (Promega). Harvested plates were subjected to a freeze-thaw cycle prior to analysis. Samples were analyzed for luciferase activity with the Dual Luciferase^®^ Reporter Assay System (Promega) on a CLARIOstar BMG Labtech luminometer, using 1 s integration per well. Experiments were performed in quadruplicate. Relative units of reporter activation were calculated as the firefly luciferase (ARE-driven) values divided by the Renilla luciferase values. All data were normalized to vehicle treatment alone.

### DE/ZI Analysis With the Script in R

The DE/ZI predicted additive effect for each dosing pair was calculated using the R code file *DEZI nearest neighbor.R*, which is included in the Supplementary Material, along with the input data from the ARE reporter assay for sulforaphane and dtBHQ. Also found in the Supplementary Material are a document with step-wise instructions, a document explaining the processes the R code follows in analyzing the data, and an Excel file to transform the returned values into a table format. Returned results from DE/ZI were graphed in Chart Studio from Plotly. The surfaces were generated by adding a mesh trace in Chart Studio.

In general, the R code follows the process outlined in Figure 3C. To generate error bars for the predicted additive effect that capture the error in the points used for the interpolations and extrapolations for each step, a Monte Carlo method is used, and iterations are conducted for each dosing pair analyzed (the default is 5,000). For each of the iterations, the data is randomly selected (using the *R* function rnorm), based on the average and standard deviation of the y value on the dosing curve. The 5,000 returned results are then averaged to give the final predicted additive effect, and the standard deviation is used as the reported error in that value. The returned result based on the means is also returned, and alternatively could be reported as the predicted additive effect. A quantitative measure of the extent of synergy generated by the R code is the FoldSynergy value, which is the actual effect divided by the predicted additive effect, with associated error propagation. We note that FoldSynergy is analogous to the inverse of the Combination Index (Eq. 2).

The Monte Carlo iterations also generate a *p*-value for whether an effect is synergistic or antagonistic. The *p*-value for synergy is equal to the number of times that the randomly generated predicted additive effect was less than the actual effect, divided by the number of randomizations. For example, if a *p*-value of 0.05 or less is returned, then 250 or fewer of the 5,000 returned results were less than the actual effect, and 4,750 or more were greater than or equal to the actual effect. We interpret this as a reasonable cut off for a synergistic interaction. If a *p*-value of 0 is returned, the *p*-value is in fact less than 1/the number of randomizations done. For example, with the default 5,000 randomizations written in the script, a value of 0 will correspond to a *p*-value < 0.0002. If a *p*-value of 1 is returned, the *p*-value is then greater than the lower limit (e.g., with 5,000 randomizations, the *p*-value is greater than 0.9998). Similarly, the *p*-value for antagonism is the number of times that the randomly generated predicted additive effect is greater than the actual effect, divided by the number of randomizations. 

**FIGURE 4:**
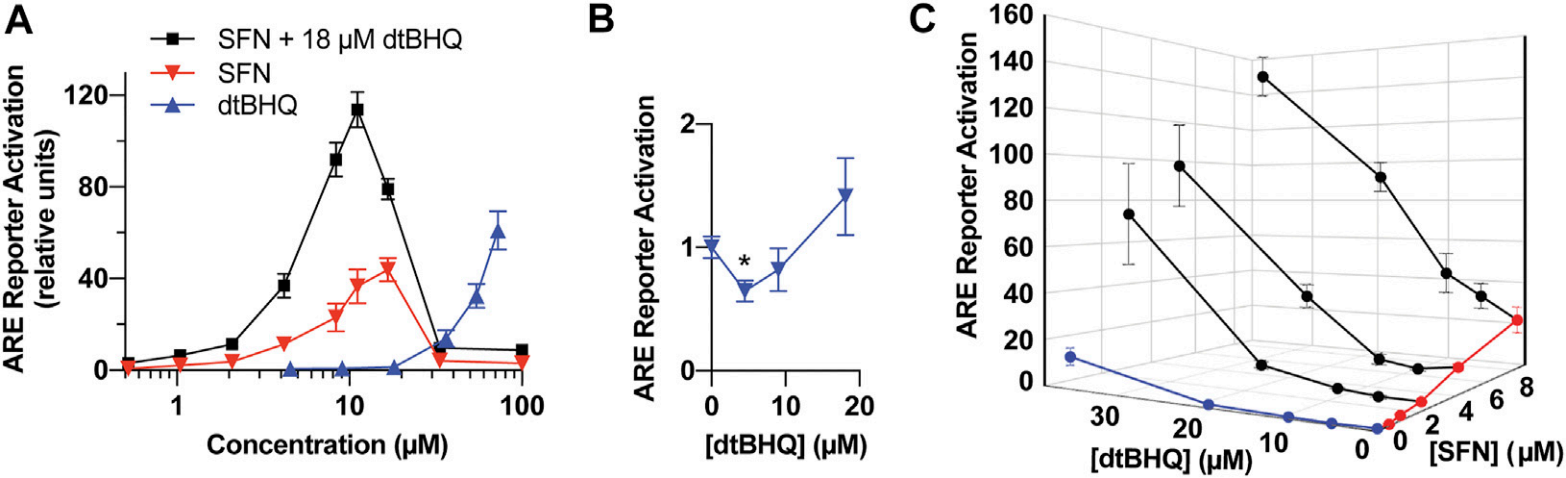
Model system for testing interactions for synergy and antagonism-a dual-luciferase ARE-reporter assay in HaCaT cells. **(A)** Full dosing curves of sulforaphane (red) and dtBHQ (blue) alone, and the same sulforaphane concentrations with a co-treatment of 18 µM dtBHQ (black). **(B)** The data from the lowest dtBHQ concentrations are plotted to show the suppression of the reporter expression at 4 µM dtBHQ (*p-value = 0.006). **(C)** Effects of various combinations of sulforaphane and dtBHQ. Combination treatment data are shown in black. The experiments were performed in quadruplicate, and the data are presented as mean ± SD.

A special case the nearest neighbor approach can accommodate is when the sign of the slope of the dosing curve changes at low doses from negative to positive, as for dtBHQ, where initially dtBHQ lowers the response below that obtained in the absence of dtBHQ (Figure 4B **)**. In areas where the slope of the curve changes, a given effect can result from either of two different concentrations. For example, for a sulforaphane ARE activation effect of 0.8, it would be possible for either of two dtBHQ concentrations to generate the same effect. Thus, a decision must be made in DE/ZI nearest neighbor as to which of two set of points will be used for interpolation to determine a dtBHQ concentration for this effect. In *DEZI nearest neighbor.R*, the points whose effects are closest in space to the effect of interest are used to determine the PAE by interpolation. In the cases where the dtBHQ concentration must be determined through extrapolation (e.g., for an effect of 0.5), the determined dtBHQ concentration that is closest to an experimental concentration used for the extrapolation is used to determine the PAE. This choice for extrapolation was fairly straightforward; as shown in Supplementary Figure S2, choosing a concentration that is furthest from one used for extrapolation does not make sense as it would then likely correspond to an effect that is much higher than in the region of interest of the dosing curve. The choice for interpolation is based on the idea that the closest interpolating pair will give the best result. However, the results may depend on the treatments that are done. We tested the effect of the interpolation choice on the results for our data by using the further data points to interpolate. The corresponding R script, *DEZI nearest neighbor max interpolation.R*, has been included in Supplementary Material. Both *R* scripts generated very similar overall conclusions.

Also provided in Supplementary Material is the *R* code *DEZI Hill equation fit.R*. The same steps are carried out as in the *DEZI nearest neighbor.R*, except that the relevant values (e.g. a_eq_) are calculated based on the results of fitting a Hill equation to the dosing curves. For example, a_eq_ is calculated based on fitting treatments of molecule one alone to a Hill equation and then solving the Hill equation with respect to the effect y of dose b to find a_eq_.

## Results

To assess for interactions of sulforaphane and dtBHQ on ARE-driven expression, a reporter assay was performed with individual titrations of sulforaphane and dtBHQ, a titration of sulforaphane in combination with 18 µM dtBHQ, and additional combinations of each compound (Figures 4A,B). Sulforaphane showed a characteristic hormetic dosing curve, with a maximal effect of 44-fold activation at 17 µM. In contrast, significant dtBHQ activation of the reporter was only observed at 36 μM, at which point toxicity was also observed (Supplementary Figure S3). In addition, dtBHQ had a suppressive effect (*p* < 0.01) on the reporter at 4 µM (Figure 4B), as previously reported (Bauman et al., 2018). Co-treating with sulforaphane and 18 µM dtBHQ, a concentration that independently produces ∼2-fold stimulation, significantly enhanced sulforaphane’s activation of the ARE reporter, up to 120-fold (Figure 4A). The substantially higher maximal activation of the reporter by the combination treatment than by either compound alone indicates a synergistic interaction between sulforaphane and 18 µM dtBHQ. As shown in Figure 4C, additional combination treatments at higher concentrations result in significantly larger effects than for any of the individual doses. At lower concentrations, there are possible antagonistic effects.

**FIGURE 5:**
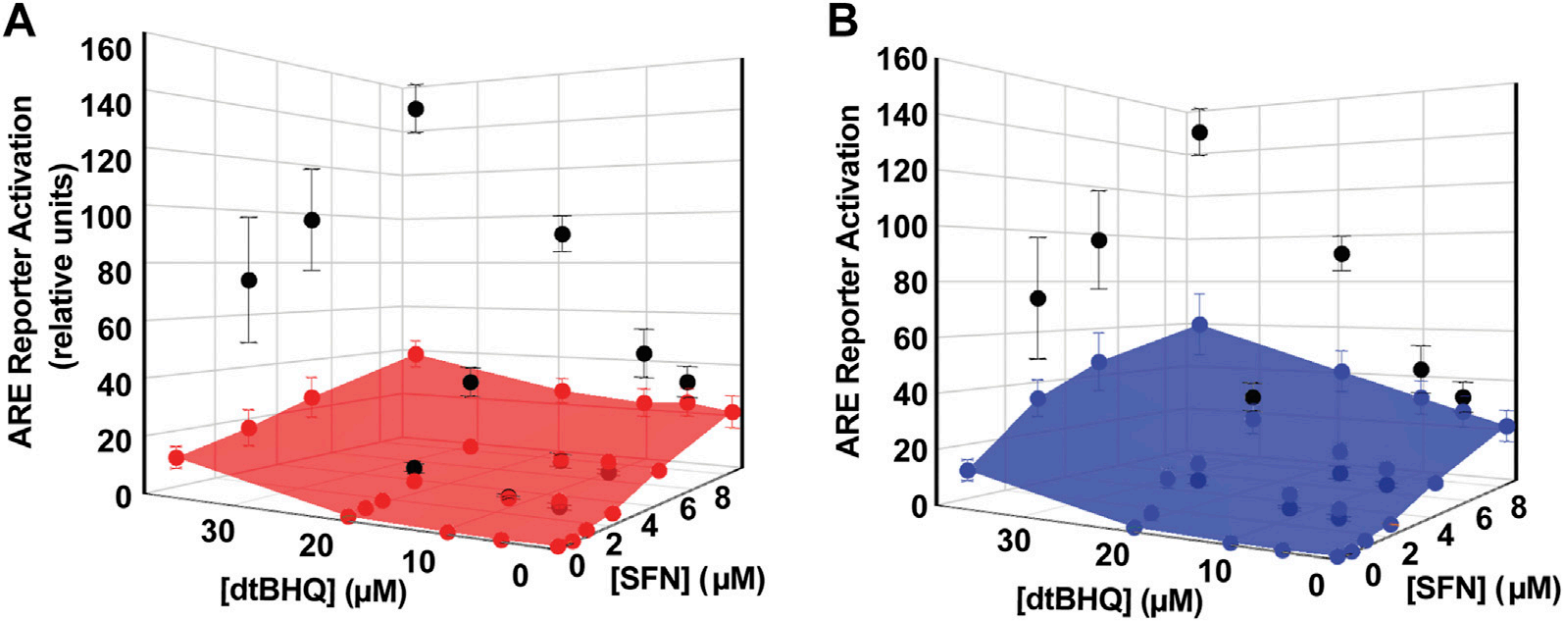
Analysis of sulforaphane-dtBHQ dosing curves and combination treatment data by the DE/ZI method. The actual effects are shown in black and replotted from Figure 3C. DE/ZI predicted additive effects interpolated from the sulforaphane dosing curve are shown in **(A)** in red and in blue in **(B)** for those predicted using the dtBHQ dosing curve. Generation of the error bars for the predicted additive results is detailed in *DE/ZI Analysis With the Script in R*.

Processing the sulforaphane and dtBHQ data set in Figure 4 using *DEZI nearest neighbor. R* results in two sets of predicted additive effects for each dosing pair, obtained using either the sulforaphane (Figure 5A) or dtBHQ (Figure 5B) individual dosing curves. Each PAE result has an associated standard deviation based on the Monte Carlo simulation. A three-dimensional surface is illustrated as a mesh that connects the PAE points in the *x*, *y*, and *z* directions. As expected, the PAE_sulf_ surface and the PAE_dtBHQ_ surface differ, because the sulforaphane and dtBHQ dosing curves do not have a constant potency ratio (see also Figure 1E). Eight of twelve of the combinations’ actual effects in Figure 5A lie above the PAE_sulf_ surface, and seven are above the PAE_dtBHQ_ surface in Figure 5B. Several actual effects at the lower sulforaphane and dtBHQ concentrations lie below both the PAE_sulf_ and PAE_dtBHQ_ surfaces. Overall, this visual result indicates that dosing pairs at higher concentrations act synergistically, while some dosing pairs at lower concentrations tend to have an antagonistic interaction.

Table 1 shows the quantitative measures of interaction for each dosing pair, with two different numerical representations of the results of the analysis. First, *p*-values are assigned for synergy and antagonism. For synergy, these are equal to the number of times that the DE/ZI randomizations generated a predicted additive effect less than the actual effect, divided by the number of randomizations. The *p*-value for antagonism is similarly calculated, using the number that generated a PAE greater than the actual effect. Second, the extent of the interaction is represented by the FoldSynergy value—the actual effect divided by the predicted additive effect. Results above one indicate synergy and those below one indicate antagonism. For example, using the sulforaphane dosing curve for interpolation, 4.5 µM dtBHQ and 2.1 µM SFN results have an antagonism *p*-value of 0.0048 and a FoldSynergy value of 0.7 ± 0.2, both of which indicate an antagonistic interaction for this dosing pair. When the same data is analyzed by DE/ZI using the dtBHQ curve to interpolate, the *p*-value is <0.0002, and the FoldSynergy value is 0.6 ± 0.2.

**TABLE 1:** Results of analyzing ARE-reporter expression data in HaCaT cells treated with SFN and dtBHQ using the R code *DEZI nearest neighbor.R*.

			DE/ZI results using the SFN curve				DE/ZI results using the dtBHQ curve			
dtBHQ (µM)	SFN (µM)	Actual effect (RU)	Predicted effect (RU)	Fold synergy	Synergy *p*-value^a^	Antagonism *p*-value^a^	Predicted effect (RU)	Fold synergy	Synergy *p*-value^a^	Antagonism *p*-value^a^
4.5	2.1	3.8 ± 0.7	5.3 ± 0.5	0.7 ± 0.2	0.9952	**0.0048**	7 ± 1	0.6 ± 0.2	1	**<0.0002**
4.5	4.2	9.1 ± 0.8	13 ± 1	0.71 ± 0.09	0.9936	**0.0064**	16 ± 2	0.62 ± 0.07	1	**<0.0002**
4.5	8.3	34 ± 6	25 ± 5	1.3 ± 0.3	0.0532	0.9468	28 ± 6	1.2 ± 0.3	0.1728	0.8272
9.1	2.1	5.2 ± 0.5	5.1 ± 0.8	1.1 ± 0.1	0.4746	0.5254	10 ± 2	0.52 ± 0.07	0.9998	**0.0002**
9.1	4.2	12 ± 2	12 ± 1	1 ± 0.3	0.7254	0.2746	21 ± 3	0.6 ± 0.2	1	**<0.0002**
9.1	8.3	40 ± 10	25 ± 6	1.8 ± 0.6	**<0.0002**	1	32 ± 7	1.4 ± 0.4	**0.0444**	0.9556
18.1	0.5	3.2 ± 0.3	2.5 ± 0.5	1.2 ± 0.1	**0.033**	0.967	5 ± 2	0.69 ± 0.07	0.8838	0.1162
18.1	1.0	6 ± 1	3.4 ± 0.5	1.9 ± 0.5	**<0.0002**	1	15 ± 3	0.4 ± 0.1	0.9944	**0.0056**
18.1	2.1	11 ± 2	7 ± 1	1.7 ± 0.3	**<0.0002**	1	18 ± 3	0.7 ± 0.1	0.9996	**0.0004**
18.1	4.2	37 ± 5	14 ± 2	2.7 ± 0.5	**<0.0002**	1	30 ± 5	1.3 ± 0.2	0.1168	0.8832
18.1	8.3	92 ± 7	27 ± 5	3.4 ± 0.4	**<0.0002**	1	42 ± 9	2.2 ± 0.2	**<0.0002**	1
18.1	11.1	114 ± 8	37 ± 6	3 ± 0.3	**<0.0002**	1	60 ± 10	2.1 ± 0.2	**<0.0002**	1
18.1	16.7	79 ± 5	45 ± 6	1.8 ± 0.1	**<0.0002**	1	63 ± 8	1.3 ± 0.1	**0.0196**	0.9804
36.3	2.1	70 ± 20	22 ± 6	4 ± 2	**<0.0002**	1	37 ± 7	2.1 ± 0.9	**0.001**	0.999
36.3	4.2	100 ± 20	29 ± 8	4 ± 1	**<0.0002**	1	50 ± 10	2 ± 0.6	**<0.0002**	1
36.3	8.3	150 ± 10	40 ± 6	3.8 ± 0.4	**<0.0002**	1	60 ± 10	2.5 ± 0.2	**<0.0002**	1

a The *p*-values for interactions that are <0.05 are in bold.

The question thus becomes how to interpret two sets of returned results for each dosing pair. In the discussion, we outline considerations for the general user in deciding how to interpret results in various scenarios. For the results with sulforaphane and dtBHQ, we consider the following test. If a given dosing pair has *p*-values indicating synergy based on interpolation for *both* of the two dosing curves, then the drugs are considered to interact synergistically at those doses, with the same logic for antagonism. By this criteria, two of the tested combinations (4.5 µM dtBHQ with 2.1 or 4.2 µM sulforaphane) show an antagonistic result, and seven show a synergistic result (primarily for 18 and 36 µM dtBHQ with 4.2 µM or higher sulforaphane).

## Discussion

**FIGURE 6:**
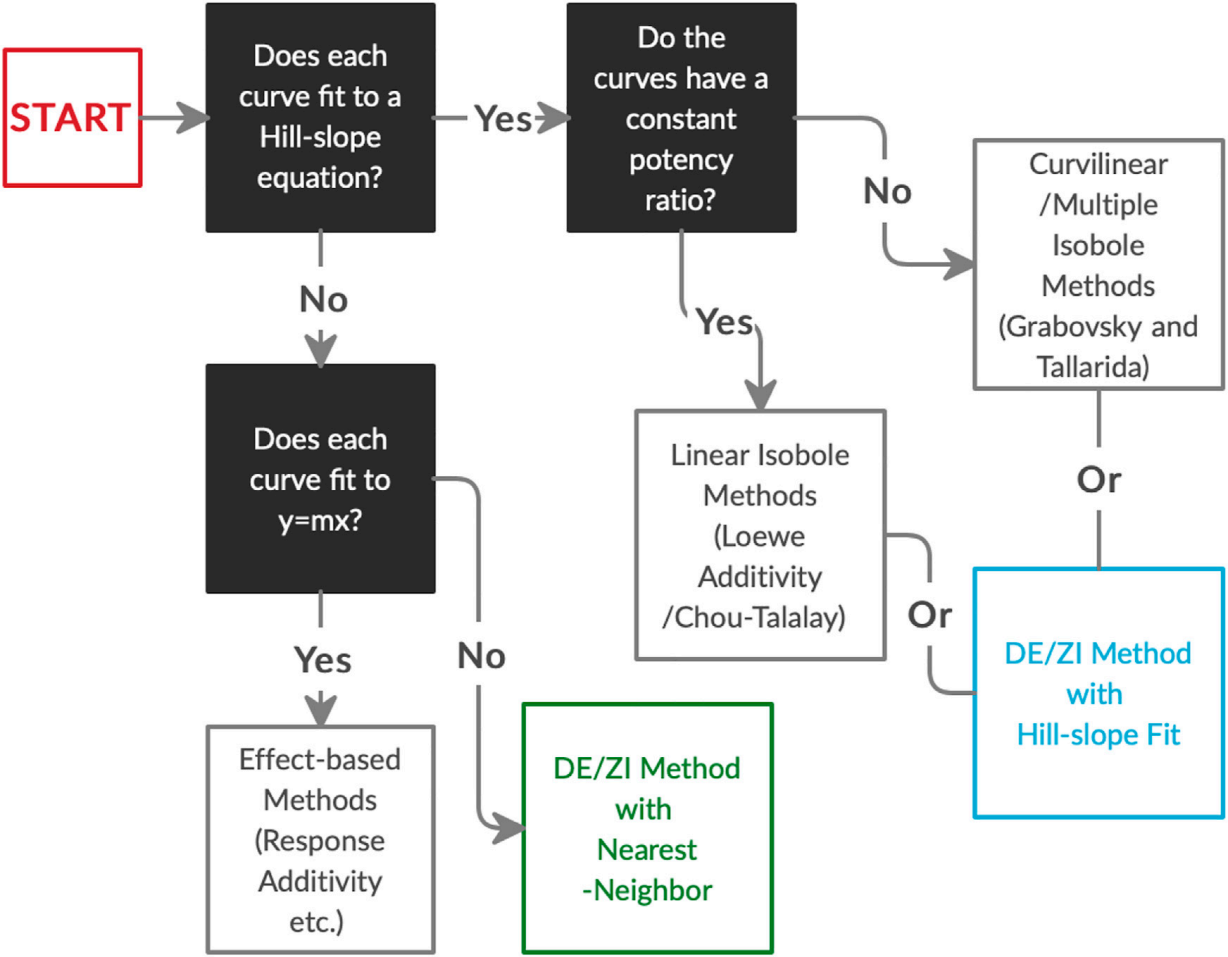
The decision process in choosing an analysis method for drug-drug interactions based on the dosing curves of the compounds. DE/ZI, as an application of Loewe additivity principles, complements existing methods, and it meets a need for analysis of non-traditional dosing curves. Associated information on constant potency ratios and effect-based methods is given in Figure 1 and *Loewe Additivity Principles Behind the DE/ZI Method and the Benefits of Releasing the Constraint of a Constant Potency Ratio*.

In order to analyze Nrf2/ARE activators for interactions, we developed the DE/ZI method, applying the principles of Loewe Additivity without assuming a constant potency ratio and using a nearest-neighbor approach. The implications of synergy and antagonism in the Nrf2/ARE pathway extend to treatment and amelioration of almost every chronic disease condition, including neurodegenerative diseases, cancer, diabetes, and cardiovascular disorders, given the central role of ARE-regulated genes in cytoprotection and metabolism. In addition to Nrf2/ARE activators, DE/ZI with nearest-neighbor allows for analysis of other drugs and phytochemicals whose dosing curves do not fit well to a Hill-slope equation, or when screening one drug with a compound library whose dosing curves are unknown. In the latter case, the dosing curve of the single drug provides predictive information about the response of the system. DE/ZI can also be used to analyze drugs with traditional Hill-slope dosing curves with or without constant potency ratios, and the R code for this analysis is included in Supplementary Material. The decision-making flow chart for analyzing drug-drug interactions (Figure 6) illustrates when to use effect-based methods, linear isobole methods such as Chou-Talalay, curvilinear multiple isobole methods, or DE/ZI for a given set of data.

DE/ZI generates a predicted additive effect, a FoldSynergy value, and associated *p*-values for every dosing combination, thus evaluating synergy or antagonism across the range of tested concentrations and allowing for the generation of a 3D response surface (Figure 5). The usefulness of response surfaces in interaction analyses is becoming appreciated, as the benefit of a combination therapy is often dependent on the doses administered (Prichard and Shipman, 1990; Keith et al., 2005; Foucquier and Guedj, 2015). A single overall assessment of synergy or antagonism for two drugs, in contrast, can miss differing interactions across the ranges of the dosing curves. In addition, given biological heterogeneity and measurement error, it is problematic to judge interactions as synergistic or antagonistic if the determination lacks associated error (Lee and Kong, 2011; Foucquier and Guedj, 2015). DE/ZI generates errors for the PAE and FoldSynergy and *p*-values through Monte Carlo-type iterations.

We note that the number of returned results from a DE/ZI analysis that show antagonism or synergy should not be taken as an indication of the overall interaction of the compounds. In other words, because more synergistic dosing pairs were found than antagonistic dosing pairs, this does not necessarily mean SFN and dtBHQ are more synergistic than antagonistic; the results depend on the range of tested concentrations. Rather, in DE/ZI, each dosing combination is separately evaluated for an interaction. A version of DE/ZI with overall assessment of interactions is under development.

The question arises: how are actual effects that fall *between* the two DE/ZI predicted additive effects assigned as synergistic or antagonistic? These effects are equivalent to those that fall in the region between two isoboles in an isobologram, a result that naturally arises from Loewe Additivity for any drug combination without a constant potency ratio. This issue was noted by Loewe himself (Loewe, 1953) and has been discussed in depth, with reasoning to consider these unassigned combinations either to be additive (Tallarida, 2007) or indeterminant (Geary, 2013). The approach that emphasizes stringency is to only consider as synergistic those dosing pairs whose actual effect is greater than both predicted additive effects, and to consider as antagonistic only those pairs whose actual effect is less than both. A second approach could be used in the case of testing a given drug in combination with a number of other compounds. In this case, a detailed dosing curve generated only for the single drug would be used to determine a PAE. Any combination with a *p*-value indicating synergy would then be tested across a range of concentrations to determine if the two molecules are synergistic across a broad 3D response surface. A third approach for treating results in the indeterminate region is based on prior knowledge about the biological system, i.e., if there is a mechanistic reason to value the dosing curve of one drug over that of the other. In general, the results from analysis of drug pair interactions in any given assay need to be examined as one part of an overall understanding to drive and guide research efforts.

By the more stringent criteria, sulforaphane and dtBHQ synergistically activate Nrf2-dependent expression at the mid-to-high end of the tested concentrations. Mechanistically how might synergy occur? The best understood biological target in this pathway is Keap1, the major repressor of Nrf2. Specifically, Keap1 C151 is a primary target of sulforaphane (Zhang and Hannink, 2003; Hu et al., 2011; Baird et al., 2013; Saito et al., 2016). Addition of sulforaphane to C151 prevents Keap1 from targeting Nrf2 for ubiquitination and degradation and allows newly synthesized Nrf2 to accumulate (Figure 2A). Thus, a primary role for sulforaphane in this pathway is to increase Nrf2 protein levels by relieving Keap1 suppression. In contrast, dtBHQ acts downstream of Keap1 cysteines and Nrf2 protein accumulation—dtBHQ did not induce Nrf2 protein accumulation on its own or increase Nrf2 levels induced by sulforaphane when they were added in combination (Bauman et al., 2018).

How then might dtBHQ signal to downstream targets in this pathway, and what might these targets be? Due to its two *tert*-butyl groups, dtBHQ itself cannot act as an electrophile upon oxidation, but redox cycles to generate other reactive species, including H_2_O_2_ (Figure 2C). The evidence supports H_2_O_2_ as the primary species responsible for synergism (Bauman et al., 2018). This was shown in part by including catalase (which reduces intracellular levels of H_2_O_2_) with sulforaphane and dtBHQ treatments, thereby suppressing protein expression of ARE-regulated genes. H_2_O_2_ is an endogenous signaling molecule with well-characterized targets in various signal transduction pathways (Marinho et al., 2014). A growing body of literature points to potential specific cysteine targets for H_2_O_2_ in the Nrf2/ARE signal transduction/activation pathway. These include C136, C71, and C124 of PTEN in the PI3K pathway (Leslie, 2006; Almazari et al., 2012; Suh et al., 2018) and C181 and C184 of H-Ras, upstream of PI3K (Oliva et al., 2003; Oeste et al., 2011). Other identified oxidative-stress responsive players in the expression of ARE-regulated genes include Bach1 (Tsuji, 2005; MacLeod et al., 2009), which competes with Nrf2 for binding to AREs, and redox sensitive microRNAs (Cheng et al., 2013). H_2_O_2_ also can induce antioxidant protein synthesis at the translational level (Wijeratne et al., 2005; Shenton et al., 2006; Grant, 2010).

We note that Keap1 cysteines, specifically C226/C613/C622/C624, are generally considered H_2_O_2_’s primary target in this pathway (Fourquet et al., 2010a; Suzuki et al., 2019). However, evidence supports the existence of other ARE-activation targets that are sensitive to lower concentrations of H_2_O_2_ than that required for Nrf2 stabilization. First, in general, fairly high concentrations of H_2_O_2_ (0.1–1 mM) are required to stabilize Nrf2 protein (Fourquet et al., 2010b; Saito et al., 2016). In our system, we tested lower concentrations of H_2_O_2_ (25 and 50 µM) that were unable to induce Nrf2 protein accumulation or enhance that of sulforaphane (Bauman et al., 2018). While these concentrations minimally activated an ARE reporter on their own (1.2 ± 0.1 and 2.6 ± 0.5 fold-activation, respectively), they highly enhanced sulforaphane’s activation of an ARE reporter (from 3.6 ± 0.6 with 2.5 µM sulforaphane alone to 8 ± 3 and 18 ± 4, respectively, with 25 and 50 µM H_2_O_2_ included). Second, seminal work by the Yamamoto lab, assigning Keap1 cysteines to specific inducers using a zebrafish embryo system, showed that factors other than Keap1 and Nrf2 were required for H_2_O_2_ to induce ARE-gene expression (Kobayashi et al., 2009). In sum, concentrations of H_2_O_2_ that are insufficient to target Keap1 cysteines have other targets in the Nrf2/ARE pathway, thereby contributing to the synergistic effect of dtBHQ and sulforaphane.

We note that experiment to experiment, while synergism is consistently observed at mid-to-high concentrations of sulforaphane and dtBHQ, antagonism at lower concentrations is not always observed for both the PAE_sulf_ and the PAE_dtBHQ_ surfaces. Analyzing our previously published ARE reporter data (Bauman et al., 2018), interactions were found to be antagonistic only when using the dtBHQ dosing curve for determinations and not when using the sulforaphane curve (Supplementary Table S1 in Supplementary Material). Thus antagonism for these two molecules is dependent on subtle changes in treatment conditions experiment to experiment. We note that the expression of the Nrf2/ARE-regulated AKR1C1 protein in response to sulforaphane was diminished by the inclusion of 5 µM dtBHQ (but significantly increased by 15 µM dtBHQ) (Bauman et al., 2018), and this effect was reproducible experiment to experiment. As to how antagonism could occur in this system, it is tempting to consider the observed suppression of Nrf2 protein synthesis by dtBHQ (Bauman et al., 2018). However, this suppression was only observed at higher dtBHQ concentrations, suggesting another mechanism is responsible. Antagonism may be due to the “dual nature” of dtBHQ as an oxidizable diphenol to also act as an antioxidant. The scavenging of superoxide by plant polyphenols is well documented (Robak and Gryglewski, 1988). The exact mechanism of how antagonism might occur is currently not clear.

For drug discovery efforts involving combination treatments, and to gain insight into mechanisms of synergy, it is essential to be able to calculate a predicted additive effect along with a measure of certainty as to whether it is greater than or less than the actual effect. DE/ZI with nearest-neighbor allows drugs with non-traditional dosing curves that do not fit to a Hill slope equation to be analyzed for interactions. For the Nrf2 pathway, future questions include the types of molecules that act synergistically or antagonistically to induce cytoprotective proteins, which of these proteins show altered expression, and the specific cysteine sensors of ROS and electrophiles that contribute to the interaction of the molecules. In addition, future versions of DE/ZI could be tailored to investigator needs in various fields, such as an overall measure of synergy or antagonism for a pair of drugs. By releasing the constraint in Loewe Additivity for a constant potency ratio and introducing the nearest-neighbor method of curve fitting, DE/ZI provides a platform for more flexibility in applying the logic of Loewe Additivity principles to analyzing diverse dosing curves.

## Data Availability

The original contributions presented in the study are included in the article/Supplementary Material; further inquiries can be directed to the corresponding authors.
