# Supplementary material for: Solving the Problem of Assessing Synergy and Antagonism for Non-Traditional Dosing Curve Compounds Using the DE/ZI Method: Application to Nrf2 Activators
Source: Front Pharmacol. 2021 Jun 7;12:686201. doi: 10.3389/fphar.2021.686201 (PMC8215699; doi:10.3389/fphar.2021.686201)
Supplement: Supplementary file 1 [file DataSheet4.docx]

**R Tutorial**

Part I: Generating a results file for your data

1. If you don’t yet have R, download the version for your computer/operating system at <https://cloud.r-project.org/> (For Windows, there will be a .exe file to download then open, and then step-by-step directions to follow. During installation, it is helpful to include an icon in the quick start menu, task bar, or desktop.)

2. Download the relevant R scripts from ___________________.

There are 2 options:

1. ***DEZI Hill equation fit.R***: For cases where the dose-response curves of each molecule both fit a Hill equation. Responses and concentrations of a molecule required for a response are determined based on the Hill equations.
2. ***DEZI nearest neighbor.R***: For any set of data where a dose response curve is available for one or both molecules. Responses and concentrations of a molecule required for a response are determined based on using existing data points, if possible, and if there is no corresponding data point, the relevant value is determined based on a linear fit of two of the nearest points.

3. Create a file with the data to be analyzed. The default is for the data file to be called data.txt. (This can be changed in the first line of the R scripts.)

The file can be generated in Excel, but it needs to be saved as a tab delimited file. An example of a formatted data file can be found at _____________.

The first column is called **blockid**. This column tells the R script which treatments are for the same pair of molecules. If you have done combinations with two different molecules (molecule A and molecule B) and then one of those molecules with another molecule (e.g. molecule A with molecule C) or even combinations of two other molecules (e.g. molecule X and molecule Y), the script can analyze each combination at the same time and generate a result for each combination of molecules. Each number in the blockid column corresponds to a single pair of molecules. For example, data from treatments with molecule A and molecule B would have a blockid entry of “1” and combinations of molecule A and molecule C would have an entry of “2”, etc. Each blockid value is treated independently for the analyses. The script is just doing all the analyses at one time. All of the results for the various blockid values will be put into the same results file. The actual results would not be any different than doing each analysis separately.

The second column is called **molecule 1**. The column corresponds to the concentrations of one of the molecules in each treatment. The next column is called **molecule 2** and is the concentration for the other molecule in each treatment. The units for concentrations of molecules 1 and 2 must be the same within a blockid. There must be treatments where the concentration of molecule 1 is 0, and there must be treatments where the concentration of the molecule 2 is 0. There must also be at least one treatment where the concentrations of both molecule 1 and 2 are not 0 (i.e. both molecules are present in that treatment).

The next column is called **actual effect**. This is the experimental result. If replicates were done, it should be the average of the replicates.

The last column is the standard deviation from the replicates and is called **sd**. If replicates were not done, enter 0 into the sd column for each treatment/row. An expected response and fold synergy will be determined; we note that the determined *p*-value in this case will not be meaningful without replicates and a corresponding standard deviation. The 2nd line of the R script can be altered to alter the number of randomizations that are done. Decreasing the number of randomizations will decrease the length of time that the R scripts run. If replicates were not done, changing the default value (5000) to 1 will make the scripts much faster and will not affect the output.

Save the file as a tab delimited text file. In Excel, select the File menu, then Save As, select the folder location you want them in, then for Save as type select Text (Tab delimited) (*.txt). Again, the default state of the script is that the file needs to be called results.txt. Put the R scripts downloaded in step 4 in the same folder as the data file.

4. Open R by clicking on the icon.

5. If you are going to run a 4-parameter log fit (Hill equation) you will need to get the drc library the first time. This is not required for the DEZI nearest neighbor.R file/method. To install the drc library, type the following into your R prompt:

install.packages('drc')

This will launch a dialogue box. The easiest CRAN to select is just the cloud (normally the first option). It might take a while to complete the process, but it only must be done once.

6. Change your working directory in R to the folder where the date file and R scripts are saved. Then to run the R script, type *source ("R script file name")* into the R prompt. For example, if you want to run the R script for the DEZI nearest neighbor.R, you would type *source ("DEZI nearest neighbor.R")* into the R prompt. Then hit return. The quotes for the file name must be "straight" quotes not “smart” (curved) quotes.

7. It may take some time for the program to run. (The example file using the DEZI nearest neighbor.R for analysis takes about 5 minutes on a Dell laptop. The length depends on the number of treatments being analyzed and which R script is being used. It also depends on the number of randomizations, which can be altered in the second line of the script. The number of randomizations though is related to how low of a *p*-value can be determined and the accuracy in determining the *p*-value, see below.)

8. The run is complete when the script creates a file called interaction_results.xls in the same folder. It is a tab delimited text file. Since it is not a true Excel file, Excel may give a warning about opening it in terms of the file format not matching the extension. Also, any program that is a text editor should also be able to open it.

Part II: Understanding the generated results file

The first column’s header is **molecule curve used for analysis**. DE/ZI predicts an expected additive effect for combination treatments between two molecules. The process of predicting an expected additive effect can use the dosing curve of either molecule for interpolation to find the equivalent doses and the predicted additive effects. The first column indicates which molecule’s dosing curve was used.

The next 5 columns are just the data given to the program in the input file (data.txt is the default) (e.g. the concentrations of the molecules, the actual effect, the standard deviation, etc.).

The 7th column (G), **mean of predicted additive effect from randomizations**, is the mean of the predicted additive effects determined from the 5000 random cases. This is a measure of the expected result in the additive case given the variation of the data. NA might be returned. This is an indication that a dose equivalence or equivalent concentration could not be determined when calculating a predicted additive effect for that treatment and so it is not possible to determine a predicted additive effect. For example, if molecule 1 reaches much higher response values than molecule 2, then the fit of the Hill equation for molecule 2 probably cannot be solved to determine a dose equivalency of molecule 2 that corresponds to treatments with molecule 1 concentrations that generate high responses, and the *DEZI Hill equation fit.R* code will return NA or NaN for such treatments. NA is also returned when one of the doses is zero. It is possible in some of the random cases that a PAE will be determined and in others it will not. In that case, the PAE based on the cases where a value is retunred will be reported.

The 8th column (H), **sd of predicted additive effect from randomizations**, is just the standard deviation associated with the mean value in the 7th column.

The 9^th^ column (I), **predicted additive effect using experimental means**, gives the predicted additive effect determined solely from the mean effects given in column E (and therefore with only one iteration). This gives an idea of the value of the predicted additive effect but doesn’t consider the variation in the data and specifically how that variation affects the possible fits of the dosing data. If this value is not similar to the value in the 7th column (G), *mean of predicted additive effect from randomizations*, that is an indication that the variation in the data allows for substantially different fits of the data that can result in different predicted additive effects. In such cases, likely some random iterations result in fits of the dosing curves that generate a substantially different predicted additive effect than that determined only from column E. In other words, the *mean predicted additive effect from randomizations* considers all the possible fits and how they affect the predicted additive value, while the predicted additive effect in the 9^th^ column does not. Like with column 7, the *DEZI Hill equation fit.R* code might return Na or NaN if a PAE cannot be determined based on the two dosing curves.

The 10th and 11th columns (J and K) give *p*-values generated from the randomizations being carried out. The 10th column, ***p*-value for synergism**, is equal to the number of cases where the randomizations generated a predicted additive value less than the actual effect divided by the number of randomizations. For example, if a *p*-value of 0.05 or less is returned, then 250 or less of the 5000 returned results were less than or equal to the actual effect, and 4750 or more were greater than the actual effect. If a *p*-value of 0 is returned, the *p*-value is really less than 1/the number of randomizations done that are not NA. The default value of randomizations written in the script is 5000, so a value of 0 will correspond in that case to a *p*-value < 0.0002. If a *p*-value of 1 is returned, that means the *p*-value is greater than the lower limit (e.g. with 5,000 randomizations, the *p*-value is greater than 0.9998). More information used to determine the *p*-values is discussed below for columns 15 to 18.

The 11th column (K), ***p*-value for antagonism**, is the same idea as the 10th column, but instead is for the randomizations that generated a predicted additive value greater than or equal to the actual effect. The same ideas related to a *p*-value of 0 and 1 apply.

We note that for the 10^th^ and 11^th^ columns, the *p*-values do not reflect an overall assessment of antagonism or synergy for the interaction of two drugs. Rather, a determined *p*-value reflects only the interaction for any single dosing combination. In other words, these *p*-values are determined without considering that multiple tests are being conducted, with a test being a combination treatment of the drugs at a single dose of each. In addition, the *p*-value is essentially a 1-tailed t-test, as opposed to a 2-tailed t-test, in that the *p*-value for synergism only asks if there is synergism, not if there is synergism and antagonism. Users should adjust the *p*-values as necessary for their purposes or conduct additional tests.

The 12th column (L), **fold synergy**, is the FoldSynergy for that treatment. The FoldSynergy is the actual experimental value divided by the mean of the expected value in the additive case from the randomizations; the 5^th^ column (E)/the 7^th^ column (G)).

The 13^th^ column (M), **error for fold synergy**, is a measure of the error in the FoldSynergy given the mean and standard deviation of the experimental value and the mean and standard deviation of the expected value. It is calculated using a standard technique for error propagations for a ratio. However, as the standard deviation for the expected value will be related to how many randomizations have been done and isn’t a true standard deviation of the measured values, the error for fold synergy is not a true standard deviation or standard error. The *p*-values that are given are a better measure of the significance of the fold synergy.

The 14th column, N, is a blank column.

The next 4 columns 15 to 18 (O-R) give information related to the *p*-value determination. When a random case is carried out, the calculated predicted additive effect can be greater than the actual case, less than the actual case, equal to the actual, or it might not be possible to determine a predicted additive effect (as discussed above for column G, the result can be NA or NaN). Information about how many randomizations generated each result are given in these four columns. The default number of randomizations is 5,000, but if some random cases might return NA or NaN for the predicted additive effect, then those random cases are not used in the determining of the *p*-value.

The 19th column (S), **number of randomizations**, is the total number of randomizations done. This should be the same as the value in the 2nd line of the R scripts. It also should be the same as the sum of the previous four columns (O-R).

The last column (T), **interpolate for mean?**, indicates if the predicted additive effect was able to be determined by interpolating from the dose curves of the molecules using the mean actual effect data (column E, the 5th column). If this was not possible, extrapolation was used. Generally, interpolation is more reliable than extrapolation.
