# Supplementary material for: Solving the Problem of Assessing Synergy and Antagonism for Non-Traditional Dosing Curve Compounds Using the DE/ZI Method: Application to Nrf2 Activators
Source: Front Pharmacol. 2021 Jun 7;12:686201. doi: 10.3389/fphar.2021.686201 (PMC8215699; doi:10.3389/fphar.2021.686201)
Supplement: Supplementary file 2 [file DataSheet3.docx]

**An explanation of how the R scripts “DEZI nearest neighbor.R”**

**and “DEZI Hill equation fit.R” work**

**Overall Goal:** Both R scripts work similarly to accomplish the same goal. They determine the predicted additive effect (PAE) for a given dosing pair of two molecules, and they determine a *p*-value for the actual effect being above (or below) that PAE.

**Summary of How They Work:**

There are four steps in DE/ZI, explained here for a generic dosing pair (a,b), meaning a dose “a” of molecule one and a dose “b” of molecule two. Also, this explanation is for the specific case of when the dosing curve for molecule one is used for interpolation of the PAE:

1) Find the effect y of the dose b of molecule two. Interpolation is used if needed.

2) Find the equivalent dose a_eq_ of molecule one for this effect of dose b, by interpolation if possible.

3) Add the actual dose a of molecule one and the equivalent dose a_eq_ of molecule one to get the total equivalent dose.

4) Determine the predicted additive effect (y) based on the total equivalent dose (a + a_eq_).

These four steps are carried out in both the DEZI nearest neighbor.R and DEZI Hill equation fit.R codes, but in different ways. In DEZI Hill equation fit.R, the relevant values (e.g. a_eq_) are calculated based on the results of fitting a Hill equation to the dosing curves. For example, a_eq_ is calculated based on fitting treatments of molecule one alone to a Hill equation and then solving the Hill equation with respect to the effect y of dose b to find a_eq_.

In the nearest neighbor approach, the code determines if any two points on the dosing curve can be used to interpolate. If yes, the two closest points will be used. Otherwise, extrapolation is used. If there are two different ways to extrapolate for a predicted additive effect, the value is calculated both ways, and then the value that is closest to the closest actual data point is selected.

For both the DEZI nearest neighbor.R and DEZI Hill equation fit.R codes, the process is iterated 5000 times for each dosing pair analyzed. Each time, the y (effect) values are randomly chosen based on the mean and standard deviation of the actual effect data, using the R function *rnorm*. These randomly selected data give a measure of the expected variation in the determined PAE, based on the variation in the actual effects, and so can be used to determine a *p*-value for the fold synergy.

**Details of how DEZI nearest neighbor.R works:**

(Below, y is always effect, x is always dose. Details are included for each line of code. The example is for the case when the dosing curve for molecule one is used for interpolation, for the generic dosing pair (a,b) for molecules one and two.)

Four steps taken in the code:

Step 1) Find y. (*Here y is* *the effect of the dose b of molecule 2. Once this is known, in step 2 an equivalent dose (a_eq_) of molecule 1 for this effect will be determined.*)

a. Take in the x, listx, and listy values (x = *the dose b of molecule 2, and the list of all doses for molecule 2 and their effects*).

b. Sort listy and listx by listx

c. Are there listx that match x? If so, report y as the mean listy value. (*When the dose b of molecule two used for the combination treatment was also used when generating the dosing curve for molecule two, the mean of the observed effects for dose b will be used*).

d. If not, find listx-x < 0 and find next listx-x > 0, if these both exist. (*This gives the two values of x from the dosing curve that flank dose b of the dosing pair (a,b). With the average of their associated y effects, this generates an interpolating pair of (x,y) data points from the dosing curve for molecule 2*). Create and solve equation of line and report y.

e. If not, will extrapolate. Determine which ends of listx (*the doses for molecule B dosing curve)* is closest to x, listx 1 (*the lowest dose)* or length(listx) (*the highest dose)*. Move in one dose on listx (to listx 2 or length(listx)-1). Create and solve equation of line and report y.

Step 2) Find x. (*Here x is the equivalent dose a_eq_ of molecule one)*

a. Take in the y, listx and listy values (*the effect of molecule 2 determined in Step 1, and the list of doses for molecule 1 and their effects*).

b. Sort listx and listy by listx

c. Are there listy that match y? If so, report x as the mean listx value. (*This is extremely unlikely but theoretically possible.)*

d. If not, find pairs of listy that trap y. If there are more than one pair, find closest pair that traps y based on minimum least squares ((y-listy_1_)^2^+(y-listy_2_)^2^)^1/2^. If same, selects 1 pair.

e. Determine equation of line for that pair and solve for x.

f. If not, must extrapolate. Find listy closest to y. Step to next listx values if possible. Solve equations of line for x. If listy closest to y is at an end with respect to x, there will be only pair. If not, and there are two pairs, return the solved x value closest to listx value for closest listy value to initial y. If same, just select one to return. *(This allows data that has a “dip”, such as the dtBHQ curve, to be handled.)*

3. Determine x, the total equivalent dose, by adding b (the dose of molecule 2) and a_eq_ (the equivalent dose of molecule one determined in step 2).

4. Find y, the predicted additive effect of (a,b), based on x from step 3, the total equivalent dose, by repeating steps a to e from step 1.
