# Supplementary material for: Solving the Problem of Assessing Synergy and Antagonism for Non-Traditional Dosing Curve Compounds Using the DE/ZI Method: Application to Nrf2 Activators
Source: Front Pharmacol. 2021 Jun 7;12:686201. doi: 10.3389/fphar.2021.686201 (PMC8215699; doi:10.3389/fphar.2021.686201)
Supplement: Supplementary file 4 [file DataSheet1.docx]

**Supplemental Figures**

**Figure 1S. Illustration of why the Response Additivity method fails for the dtBHQ dosing curve. (A)** The actual effects of doses of dtBHQ, used to determine the DE/ZI predicted effects shown in B and C. The inset shows the dip at low concentrations. The dashed line is explained below. **(B and C)** Comparison of the DE/ZI Predicted Additive Effect to the Response Additivity Predicted Effect when combining dtBHQ with itself, a Sham Combination test. The concentrations of dtBHQ are shown on the x and y axes, and the predicted additive effects (PAEs) are plotted on the z axis. Graphs B and C differ only in that the z-axis in C is on a log scale to illustrate fold differences for each dosing pair. The DE/ZI PAE is the actual effect of the total dose—for example, at the dosing pair (18,18), the predicted effect is the actual effect at 36 µM dtBHQ, 13.8 RU (shown in (A)). In contrast, the Response Additivity PAE is calculated at each dose of dtBHQ as the value of the effect plus that same value. For example, the Response Additivity Predicted Effect at the dosing pair of (18, 18) equals the value of the effect of the individual dose of 18 µM dtBHQ plus that same value: 1.4 RU + 1.4 RU = 2.8 RU. (The predicted additive effect for both methods at (0,0) is set to 1 RU, since all data is normalized to the vehicle only control.) The only similar result for the two methods is for the (9, 9) dosing pair, which as shown in (A) is the only combination where the line connecting the point (0,0), the point for effect of the dose 9 (9, 0.8), and the point for the effect of the doubled dose 18 (18, 1.5) form a straight line that fit reasonably well to y=mx (shown as a dashed line). As shown in Figure 3 in the main text, only data that fit to y=mx will pass the Sham Combination test.


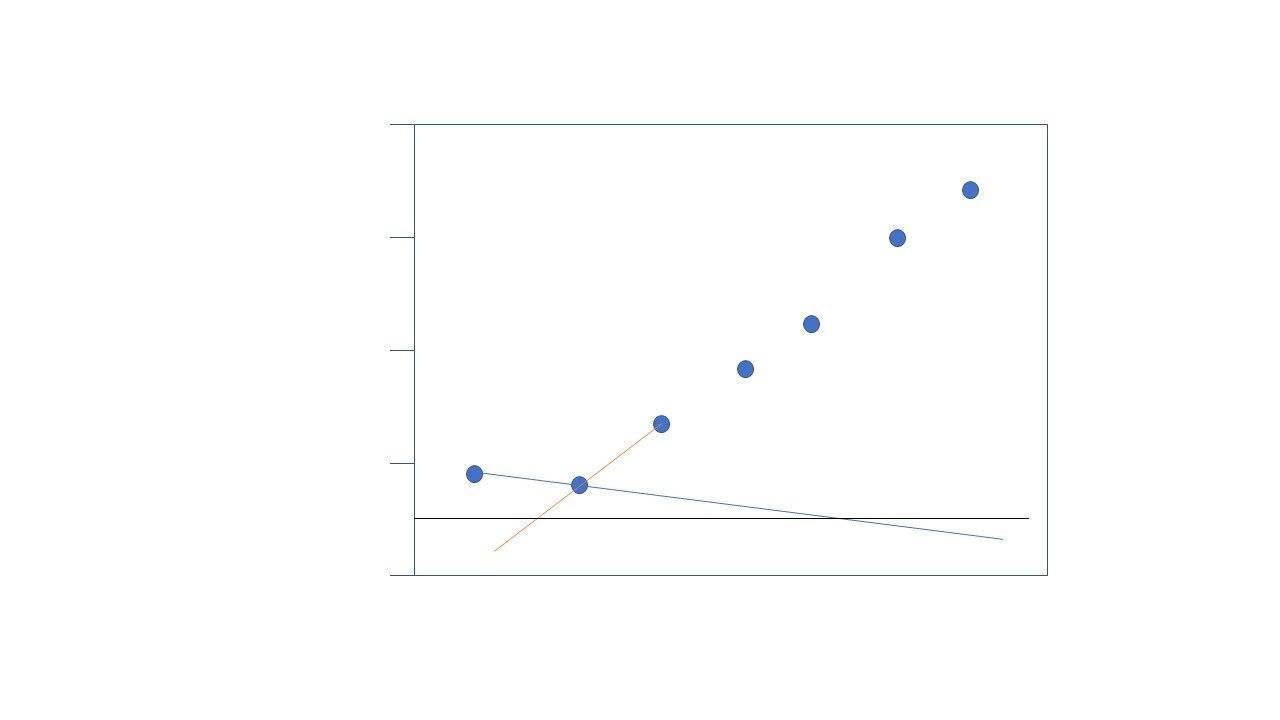


**Figure 2S.** An explanation of how DE/ZI nearest neighbor accounts for a dip in a dosing curve when extrapolation is required. A mock dosing curve is shown, with dose on the x-axis and effect on the y-axis. The effect to be extrapolated is shown by the black line. This effect could be extrapolated from either the blue line or the orange line. Given the y-value for the black line, the intersection of the blue and black line does not represent a reasonable x-value to generate that y-value.  The intersection of the orange and the black line is a much more reasonable estimate of the x-value needed to generate the y-value of the black line.  Thus, the R code takes the x value closest to the points used for extrapolation.


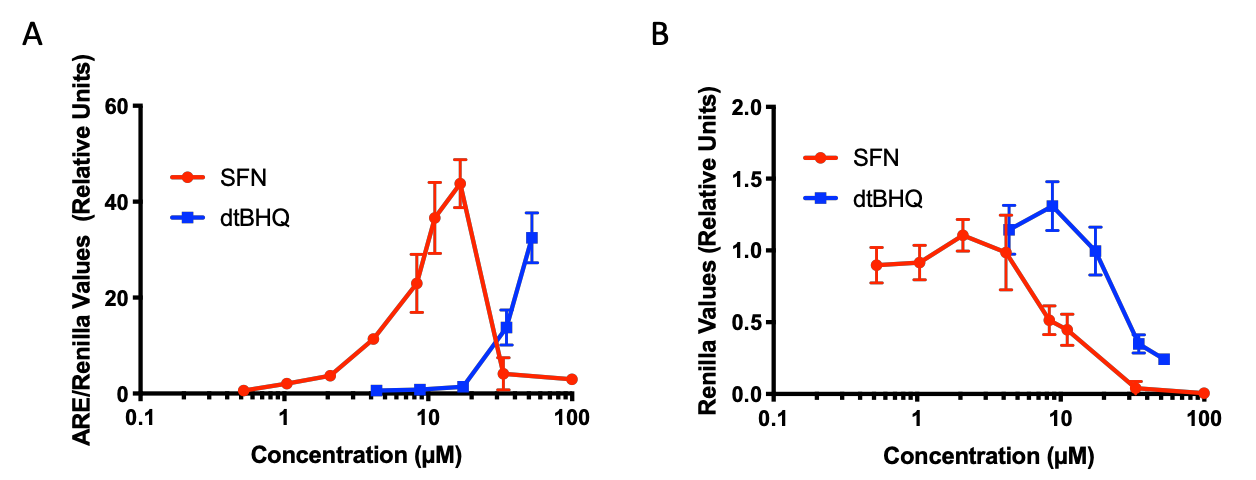


**Figure 3S.** ARE reporter assay results for sulforaphane and dtBHQ individual dosing curves. This dual luciferase assay is reported as the ARE firefly luciferase values divided by the Renilla luciferase values (A), where the Renilla luciferase is under the control of a constitutively active promoter. The Renilla values alone (B) are reflective of global translation and thus cell health. Both dtBHQ and sulforaphane show cytotoxicity at concentrations where the ARE/Renilla values are still increasing. The experiment was performed in quadruplicate and the data are presented as mean ± standard deviation.

**Supplemental Table**

**Table S1.** Results of analyzing ARE-reporter expression data in HaCaT cells treated with SFN and dtBHQ from (Bauman et al., 2018) using the R code *DEZI nearest neighbor.R*.

|  |  |  | **DE/ZI Results using the SFN Curve** | | | | **DE/ZI Results using the dtBHQ Curve** | | | |
| --- | --- | --- | --- | --- | --- | --- | --- | --- | --- | --- |
| **dtBHQ (µM)** | **SFN (µM)** | **Actual Effect (RU)** | **Predicted Effect (RU)** | **Fold Synergy** | **Synergy  *p*-value^a^** | **Antagonism *p*-value^a^** | **Predicted Effect (RU)** | **Fold Synergy** | **Synergy  *p*-value^a^** | **Antagonism *p*-value^a^** |
| 3.1 | 2.5 | 3.5 ± 0.5 | 3.3 ± 0.8 | 1.1 ± 0.2 | 0.4258 | 0.5742 | 5 ± 1 | 0.7 ± 0.1 | 0.921 | 0.079 |
| 6.3 | 2.5 | 5 ± 2 | 3.4 ± 0.8 | 1.6 ± 0.7 | **0.0032** | 0.9968 | 6 ± 2 | 0.9 ± 0.4 | 0.683 | 0.317 |
| 12.5 | 0.1 | 2 ± 1 | 1.6 ± 0.4 | 1 ± 1 | 0.0742 | 0.9258 | 4 ± 1 | 0.6 ± 0.5 | 0.8046 | 0.1954 |
| 12.5 | 0.4 | 2.12 ± 0.06 | 1.9 ± 0.5 | 1.14 ± 0.04 | 0.2702 | 0.7298 | 5 ± 1 | 0.45 ± 0.02 | 0.9838 | **0.0162** |
| 12.5 | 1.2 | 3.2 ± 0.4 | 2.7 ± 0.9 | 1.2 ± 0.2 | 0.2564 | 0.7436 | 6 ± 2 | 0.6 ± 0.1 | 0.952 | **0.048** |
| 12.5 | 2.5 | 12 ± 3 | 5 ± 1 | 2.6 ± 0.9 | **< 0.0002** | 1 | 10 ± 3 | 1.3 ± 0.4 | 0.1786 | 0.8214 |
| 12.5 | 3.7 | 16 ± 2 | 7 ± 2 | 2.2 ± 0.4 | **0.0002** | 0.9998 | 12 ± 3 | 1.3 ± 0.2 | 0.1306 | 0.8694 |
| 12.5 | 7.0 | 39.7 ± 0.9 | 16 ± 3 | 2.46 ± 0.08 | **< 0.0002** | 1 | 20 ± 4 | 1.95 ± 0.06 | **< 0.0002** | 1 |
| 12.5 | 11.0 | 77 ± 4 | 33 ± 5 | 2.3 ± 0.2 | **< 0.0002** | 1 | 37 ± 6 | 2.1 ± 0.2 | **< 0.0002** | 1 |
| 25.0 | 2.5 | 41 ± 5 | 10 ± 2 | 4.1 ± 0.6 | **< 0.0002** | 1 | 16 ± 5 | 2.5 ± 0.4 | **< 0.0002** | 1 |
| 50.0 | 2.5 | 70 ± 20 | 28 ± 7 | 3 ± 1 | **< 0.0002** | 1 | 30 ± 10 | 2.4 ± 0.9 | **< 0.0002** | 1 |

^a^The *p*-values for a synergistic interaction that are < 0.05 are colored green, and the *p*-values for an antagonistic interaction that are < 0.05 are colored blue.
